# Effect of local administration of microRNA-31/210 on bone regeneration surrounding hydroxyapatite/tricalcium phosphate -coated titanium implant in an ovariectomized rat model

**DOI:** 10.1016/j.reth.2025.101055

**Published:** 2025-12-20

**Authors:** Shinichi Ueki, Takeshi Shoji, Hideki Saka, Hiroki Kaneta, Hiroyuki Morita, Yosuke Kozuma, Nobuo Adachi

**Affiliations:** a Department of Orthopaedical Surgery, Graduate School of Biomedical Sciences, Hiroshima University, 1-2-3 Kasumi, Minami, Hiroshima 734-8551, Japan; b Department of Orthopaedical Surgery, Saka Midorii Hospital, 6-28-1 Midorii, Asaminami, Hiroshima 731-0103, Japan

**Keywords:** microRNA, microRNA-31, MicroRNA-210, Ovariectomized rat model, Titanium implant, Osseointegration

## Abstract

**Background:**

With the aging population, the prevalence of total joint arthroplasty in older adults with compromised bone conditions, such as osteoporosis, is increasing, raising concerns on the initial fixation of implants and aseptic loosening. Recent studies have highlighted the potential of microRNAs (miRNAs) to enhance osteogenesis and angiogenesis, potentially improving implant osseointegration. This study aimed to identify miRNAs with the highest osteogenic and angiogenic potential in vitro, and evaluate its effects on implant osseointegration and surrounding bone regeneration in an ovariectomized (OVX) rat model.

**Methods:**

In vitro studies were conducted to identify miRNAs exhibiting the greatest osteogenic and angiogenic potential among candidate miRNAs (miR-31, -34a, −146, −210, −218, and −31 + 210). Subsequently, the most effective miRNA was selected and locally administered to the bone matrix, where hydroxyapatite/tricalcium phosphate (HA/TCP)-coated titanium implants were placed in the femurs of OVX rats for in vivo studies. At 2, 4, and 8 weeks post-implantation, implant osseointegration, osteogenesis, angiogenesis of the matrix bone, and the initial fixation of the implant were evaluated using histological, genetic, radiological, and biomechanical assessments.

**Results:**

miR-31 and miR-210 were strongly associated with osteogenesis, whereas miR-31 was strongly associated with angiogenesis. Moreover, the simultaneous administration of miR-31 and miR-210 resulted in the highest osteogenic potential among the miRNAs tested. In the OVX rat model, local administration of miR-31 + 210 significantly enhanced implant osseointegration, osteogenesis, angiogenesis within the bone matrix, and initial fixation of the implant compared to controls.

**Conclusion:**

Local administration of miR-31 + 210 around HA/TCP-coated implants effectively improved implant osseointegration, the bone matrix environment, and initial fixation of implants in osteoporotic bone, likely by promoting osteogenesis and angiogenesis. This strategy holds promise as a novel regeneration therapy for enhancing implant fixation in patients with poor bone quantity.

## Highlights

• miR-31 and miR-210 synergistically drive osteogenesis and angiogenesis.
• Local miR-31 + 210 administration accelerates osseointegration in osteoporotic bone.
• Enhanced peri-implant bone regeneration and mechanical implant fixation shown.
• Novel miRNA-based regenerative therapy to improve the bone environment around implant.

## 1 Introduction

Total joint arthroplasty (TJA) is among the most successful surgical interventions in modern medicine, offering substantial improvement in the patient's quality of life [1,2]. However, with an increasingly aging population, TJAs are being performed more frequently in individuals with osteoporosis, revision arthroplasty, post-infection, and other conditions associated with bone loss and reduced bone quality. Aseptic loosening remains the leading cause in revision arthroplasty for TJA [[3], [4], [5]], and has been linked to risk factors, such as obesity, hyperactivity, smoking, osteoporosis, and low bone quality [[6], [7], [8]]. Considering the prevalence of osteoporosis among patients undergoing TJA [9,10], the incidence of aseptic loosening triggered by osteoporosis is expected to increase. Studies demonstrated that reduced bone quantity and compromised matrix quality impair osseointegration to the implant [11], and that initial implant migration is associated with decreased long-term survival [12]. Therefore, improving the initial fixation strength of implants and surrounding bone regeneration in patients with low bone quantity remains a critical challenge to be addressed.

Osteoporosis medications are generally used following arthroplasty to improve implant fixation and enhance bone mass and quality of the matrix bone. Although they reduce the risk of aseptic loosening and improve long-term survival rates [13], their localized effects, particularly in long bones, require a prolonged period to manifest [14,15]. Therefore, a novel regeneration therapy is necessary to increase the bone volume around implants and improve the fixation force of the implants during the early postoperative period. Therefore, we focused on the potential role of microRNA (miRNA), a short non-coding RNAs that act as repressors of gene expression at the post-transcriptional level [16]. miRNA bind to the 3′-UTR regions of mRNA of target genes, and induce mRNA degradation or inhibit protein translation, subsequently regulating gene expression. Differential regulation of miRNAs can simultaneously regulate the endogenous expression of multiple growth factors [17]. Recently, miR-31, -34a, −146, −210, and-218 have been identified as promoters of osteogenesis and angiogenesis [[18], [19], [20], [21], [22]], and the local application of miRNA as an implant coating have been reported in the literature [[23], [24], [25]]. However, the most effective miRNA for promoting osteogenesis and angiogenesis, along with the efficacy of combining miRNAs with osteogenic and angiogenic potential, remain unresolved.

The primary objective of this study was to identify miRNAs that exhibit the greatest osteogenic and angiogenic potential. Subsequently, we evaluated the effects of locally delivering the selected miRNA to the bone matrix around hydroxyapatite/tricalcium phosphate (HA/TCP)-coated titanium implants on osseointegration and bone regeneration in an ovariectomized rat model, providing insights into potential therapeutic strategies to improve implant fixation in osteoporotic bone.

## 2 Material and methods

All procedures were performed in accordance with the Guidelines for Animal Experimentation of Hiroshima University, with the approval of the Committee of Research Facilities for Laboratory Animal Sciences, Graduate School of Biomedical Sciences, Hiroshima University [A22-131]. As this study involved animal experimentation, a clinical trial number is not applicable.

### 2.1 In vitro study

#### 2.1.1 Cell culture

Human bone marrow-derived mesenchymal stem cells (hMSCs) (LONZA, Basel, Switzerland) obtained from five donors were pre-cultured in hMSC culture medium (StemPro MSC SFM, Invitrogen, Carlsbad, CA, USA) with antibiotics. Similarly, human umbilical vein endothelial cells (HUVECs) (Lonza Group AG, Basel, Switzerland) were precultured in endothelial basal medium-2 (Lonza, Basel, Switzerland). Both cell types were maintained in a humidified environment with 5 % CO2 at 37 °C. For subsequent in vitro experiments, each condition was repeated independently five times, and the results were aggregated for statistical analysis.

#### 2.1.2 Transfection of miR-31, 34a, 146, 210, 218, 31 + 210 mimics into MSCs

A total of 2 × 10^4^ hMSCs were seeded in 24-well plates overnight, after which the medium was replaced with an osteogenic medium (StemPro Osteogenesis Differentiation Kit, Invitrogen). Following osteogenesis induction, MSCs were transfected with 50 nM miR-31 (sequences 5′-AGGCAAGAUGCUGGCAUAGCUG-3′), miR-34a (sequences 5′-UGGCAGUGUCUUAGCUGGUUGU-3′), miR-146 (sequences 5′-UGAGAACUGAAUUCCAUGGGUU-3′), miR-210 (sequences 5′-AGCCCCUGCCCACCGCACACUG-3′), miR-218 (sequences 5′-UUGUGCUUGAUCUAACCAUGU-3′), or a combination of miR-31 and miR-210 (miR-31 + 210, comprising 25 μM each) mimics, as well as Silencer Negative Control siRNA#1 (Thermo Fisher Scientific, Waltham, Massachusetts, USA) as control, using Lipofectamine-RNAi MAX (Invitrogen) in accordance with the manufacturer's protocol. miRNAs with previously documented osteogenic and angiogenic effects [[18], [19], [20], [21], [22]] were selected for this study. Following a 10-min incubation period at room temperature, the mixture was transferred into 24-well plates and incubated at 37 °C in a humidified air containing 5 % CO2. The cells were maintained under osteogenic differentiation conditions for 14 days, with the osteogenic medium refreshed every 3 days. After this period of osteogenic induction, the cells underwent analyses for calcification and gene expression.

#### 2.1.3 Alizarin red staining

To detect calcification during differentiation, cells were fixed with 4 % paraformaldehyde (PFA) for 15 min 14 days post-transfection. Subsequently, the cells were washed with distilled water, stained with 1 % alizarin red staining solution for 20 min, and washed again to remove excess dye. Cell images were acquired using a microscope and the stained area was quantified through binarization using the Auto Threshold in ImageJ (NIH). The thresholding algorithm employed in the auto-threshold is Otsu.

#### 2.1.4 Introduction of miR-31, 34a, 146, 210, 218, 31 + 210 mimics into HUVECs

HUVECs were seeded at a density of 1.0 × 10^4^ cells/well in 96-well plates pre-coated with the Matrigel Matrix (Merck Millipore, Billerica, MA, USA). Similar to MSCs, 50 nM of miR-31, 34a, 146, 210, 218, 31 + 210 (comprising 25 μM each) mimics, or with equivalent concentrations of vascular endothelial growth factor (VEGF) and siRNA ware introduced to the bottom well of the multiwall insert assembly in 750 μl of serum-free medium. The cells were incubated at 37 °C with 5 % CO2. At 8 h post incubation, branch points were counted following a previously described method [26] to evaluate the effect of each miRNA on angiogenesis. Additionally, the total tube length was measured using ImageJ software.

#### 2.1.5 Quantitative real time polymerase chain reaction

To assess the osteogenic potential of each transfected miRNA, the expression levels of collagen type 1A1 (COL1A1) and runt-related transcription factor 2 (Runx2) were analyzed using real-time polymerase chain reaction (PCR) on post-transfection day 14. Cells were harvested and total RNA was extracted using TRIzol (GIBCO BRL, Palo Alto, CA, USA) according to the manufacturer's instructions. Quantitative real-time PCR was conducted using TaqMan mRNA assay kit for COL1A1, Runx2, and GAPDH. Total RNA (1 μg) was reverse transcribed using SuperScript VIVO Master Mix (Invitrogen). Quantitative real-time PCR was performed using the TaqMan Fast Advanced PCR Master Mix (Applied Biosystems, Foster City, CA, USA) in conjunction with a StepOne and Biosystems Real-time PCR System (Applied Biosystems). The expression of Runx2 and COL1A1 was evaluated at post-transfection day 14 and was assessed relative to the expression of GAPDH. A threshold cycle (Ct) was observed in the exponential phase of amplification and relative expression levels were quantified using standard curves for target genes and endogenous controls. Geometric means were used to calculate the delta–delta Ct (ΔΔCt) values and were expressed as 2^−ΔΔCt^. Each control sample was set to a value of 1, and the fold-change in the target genes was calculated using this value as a reference.

### 2.2 In vivo study

#### 2.2.1 Animal

Twelve-week-old female Sprague Dawley (SD) rats (Charles River Laboratories, Tokyo, Japan) were used in these experiments. Rats were housed at the Laboratory Animal Center of Hiroshima University under standard diurnal light/dark conditions. They were provided with a standard commercial diet and tap water and libitum.

#### 2.2.2 Ovariectomy procedures

Bilateral ovariectomy (OVX) or or sham operation was performed on twelve-week-old female SD rats. Inhalational anesthesia was induced and maintained using 2 % isoflurane by volume. Following anesthesia, the rats were positioned in a lateral orientation. The ovary was located in an adipose pad immediately beneath the muscles and excised by cutting above the clamped area, while the uterine horn was returned to the abdomen. After confirming hemostasis, the peritoneum, muscle layers, and skin were closed with absorbable sutures. Postoperatively, the animals were monitored for signs of pain, infection, and activity levels. 4 weeks after OVX and or sham operation (both group n = 4), the baseline bone volume-to-tissue volume ratio (BV/TV) of the distal femur was measured using micro-CT, with the region of interest (ROI) settings aligned with those detailed in existing reports [27].

#### 2.2.3 Preparation of miR-31 + 210 and siRNA-Atelocollagen complex

In the vivo study, miR-31 + 210 was selected for local administered based on its superior osteogenic and angiogenic potential demonstrated in vitro study. Synthetic mimics of miR-31 (sequences 5′-AGGCAAGAUGCUGGCAUAGCUG-3′) and miR-210 (sequences 5′- AGCCCCUGCCCACCGCACACUG-3′) were obtained from Hokkaido System Science (Sapporo, Japan) and used for local delivery in the experimental group. Additionally, double-stranded RNA (dsRNA) molecules lacking specific function (sequences 5′-ATCCGCGCGATAGTACGTA-3′ and 3-overhang dTdT/dTdT [sense/antisense] siRNA-negative control; B-Bridge International) were used as a control. Atelocollagen a solution of highly purified type I collagen isolated from the calf dermis through pepsin treatment (Koken, Tokyo, Japan) was used as the delivery system for the miRNAs and siRNAs. Our prior research highlighted atelocollagen as a miRNA carrier, providing sustained release and efficient microRNA uptake into target cells [28,29]. Additionally, it exhibits high transfection efficiency, facilitating the uptake of microRNA into osteoblasts and chondrocytes, and has been reported to enhance osteogenic and cartilage formation [30,31]. This delivery system ensures localized retention and cellular delivery of miRNAs. The complex of miR-31 + 210 and atelocollagen was prepared by combining 12.5 μl atelocollagen, 2.5 μl sterile water, and 5.0 μl of each miRNA mimic solution (20 mg/15 mL) to achieve a final concentration of 10 μM for each miRNA. Subsequently, the complex was mixed by rotation at 4 °C for 20 min. The siRNA–atelocollagen complex was prepared using the same protocol.

#### 2.2.4 Implant placement procedures

Implant placement surgery was performed 4 weeks after OVX. The procedure was performed under standard aseptic conditions, using a previously described anesthetic protocol. Following a skin incision on the distal lateral femur, the vastus lateralis femoris was separated and the distal lateral femur was exposed. A bone groove 6 mm in length, 3 mm in width, and 2 mm in depth was created using a surgical air tome. HA/TCP-coated titanium implants measuring 5 mm in length, 2 mm in width, and 3 mm in depth were inserted into the groove. The study was divided into two distinct groups: one group received a single local administration of miR-31/miR-210–atelocollagen complex, with each miRNA co-administered at final concentration of 10 μM, into the matrix bone of the implant insertion site (miR group), while the other group received local administration of non-functional siRNA (siR group). These implants were fabricated from an actual hip-prosthesis stem (VerSys HA/TCP Fiber Metal Taper; Zimmer Biomet, Zimmer Biomet Inc., Warsaw, IN, USA) and cut using wire electrical discharge machining at a specialized facility (KSI, Shizuoka, Japan). This implant is a titanium fiber mesh made of titanium alloy (Ti-6Al-4V) to which HA/TCP has been applied using a plasma spray technique. The ratio of HA to TCP in the HA/TCP coating is 65 %/35 % [32]. After confirming the press fit of the implant, the wound was closed in layers. Unprotected weight bearing was permitted immediately in both groups. Postoperatively, the animals were monitored for signs of pain, infection, and activity levels. Rats were euthanized at 2, 4, and 8 weeks post-implantation, and histological, genetic, radiological, and biomechanical assessments were conducted at each time point (n = 5 per group). At the study endpoint, all rats were humanely euthanized under deep anesthesia to minimize pain and distress. Anesthesia was induced with 4–5 % isoflurane in an induction chamber and maintained with 2–3 % isoflurane via a nose cone until loss of pedal reflex was confirmed. While under deep anesthesia, animals were euthanized by exsanguination via cardiac puncture.

#### 2.2.5 Histological analysis

**Fig. 1:**
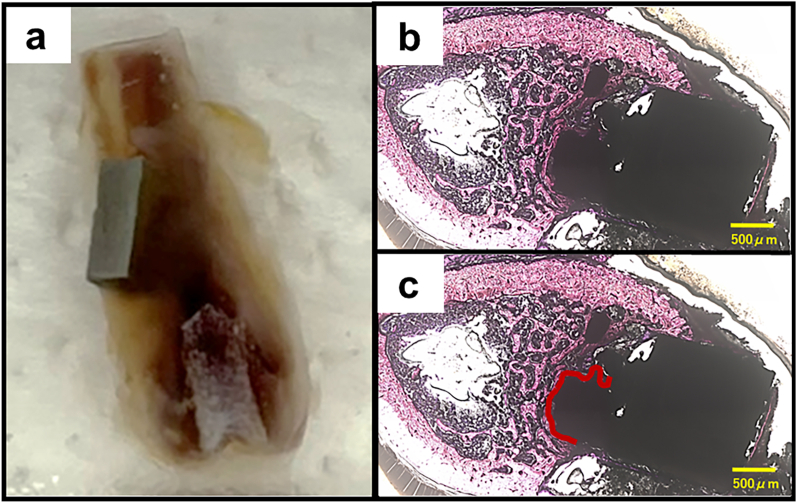
Histological analysis.

Post-sacrifice, the femur containing the implanted device was excised and tissue blocks were procured. These blocks were dehydrated using a series of increasing ethanol concentrations and subsequently embedded in light-polymerized polyester resin (Technovit 7200VLC, Heraeus Kulzer, Wehrheim, Germany). Complete polymerization was achieved using photopolymerization equipment (BS5000, EXAKT Apparatebau, Norderstedt, Germany), and the specimens were sectioned with a high-precision diamond disk to yield cross-sections with a thickness of 200 μm. The undecalcified specimens were further reduced to approximately 100 μm in thickness using a grinding machine (MG5000, EXAKT Apparatebau, Chemnitz, Germany). The sections were subjected to hematoxylin and eosin (HE) staining and examined using a digital microscope (BZ-9000; Keyence, Osaka, Japan). To evaluate osseointegration, the bone implant contact (BIC) was quantified as the percentage of the direct bone-implant interface length relative to the total bone length (Fig. 1).

#### 2.2.6 Quantitative real time polymerase chain reaction

Intrinsic factors in the trabecular bone around the implant in rats were detected using real-time PCR. Total RNA was extracted from the trabecular bone around the implant using Isogen reagent (Nippon Gene) and an RNA purification kit (Direct-zol RNA microprep, Zymo Research). Complementary DNA was synthesized using a Reverse Transcription system (iScript Supermix, Bio-Rad) according to the manufacturer's protocol. Real-time PCR was performed using TaqMan Gene Expression Assay probes (Thermo Fisher Scientific) for COL1A1 (Rn01463848m1), RUNX2 (Rn01512298m1), and VEGF (Rn0511601m1) in the trabecular bone around the implant. GAPPH (Rn01749022m1) was used as an internal control to normalize sample differences. The ΔΔCt method was used to analyze real-time PCR data. The expression levels of COL1, RUNX2, and VEGF in the cancellous bone matrix at the time of implantation were used as controls. Expression levels were subsequently measured, and fold changes relative to the control were evaluated at 2, 4, and 8 weeks following the administration of miRNA or siRNA. This analysis aimed to assess the impact of local miRNA administration on the enhancement of osteogenesis and angiogenesis in the cancellous bone surrounding the implant.

#### 2.2.7 Radiological analysis

**Fig. 2:**
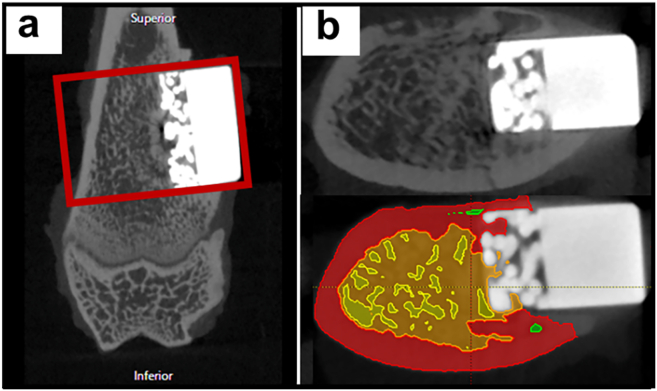
Radiological analysis.

Femoral samples were analyzed under high-resolution micro-CT (Cosmo Scan GXⅢ; Rigaku Corporation, Tokyo) using the following parameters: X-ray voltage = 100 kV; X-ray tube current = 120 μA; and exposure time = 4 min. Beam-hardening artifacts were reduced using copper (0.1 mm) filters. Prior to analysis of the bone microstructure, raw images were reconstructed using Cosmo Scan GX Image Analysis Software (Rigaku Corporation) with an isotropic voxel size of 5.5 μm. The microstructural parameters of the femurs were assessed using the Analyze 15.0 software (Analyze Direct, Inc., KS, USA). The ROI was aligned with the longitudinal axis of the implant, and the BV/TV was quantified within this specified area (Fig. 2).

#### 2.2.8 Biomechanical analysis

**Fig. 3:**
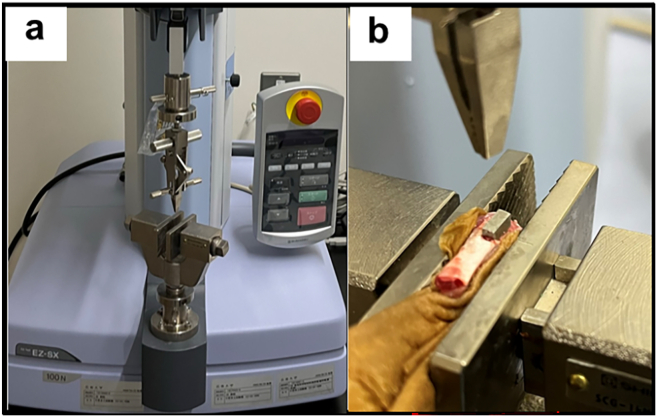
Biomechanical analysis.

To evaluate the strength of implant fixation, a pull-out test was conducted as a biomechanical assessment using a uniaxial material testing system (EZ-SX; SHIMADZU, Japan) to measure the tensile strength under a 100 N load. Both ends of the femoral specimen and implant were secured in clamps with a wet kimtowel, and the specimen was subjected to tension parallel to the long axis of the implant at a constant strain rate of 1 mm/min (Fig. 3). This process was terminated when the peak force was reached, which is indicative of implant loosening. This peak value was recorded in newtons (N) as the maximum pull-out force. Biomechanical assessments were conducted at two and four weeks post-implantation. This timing was selected as callus formation was observed on the implant surface by eight weeks, which was deemed to potentially impede the accurate measurement of the pull-out strength of the implant and cancellous bone.

### 2.3 Statistical analysis

Statistical analyses were performed using the EZR software (Saitama Medical Center, Jichi Medical University, Saitama, Japan), which is the graphical user interface for R (The R Foundation for Statistical Computing, Vienna, Austria). Statistical analysis of the in vitro study was performed using the Kruskal–Wallis test with Steel-Dwass correction. Furthermore, statistical analysis of the in vivo study was carried out between the two groups using the Mann–Whitney *U* test. All data are presented as mean ± standard deviation (SD), and a p-value <0.05 was considered statistically significant.

## 3 Result

### 3.1 In vitro study

#### 3.1.1 Alizarin red staining

**Fig. 4:**
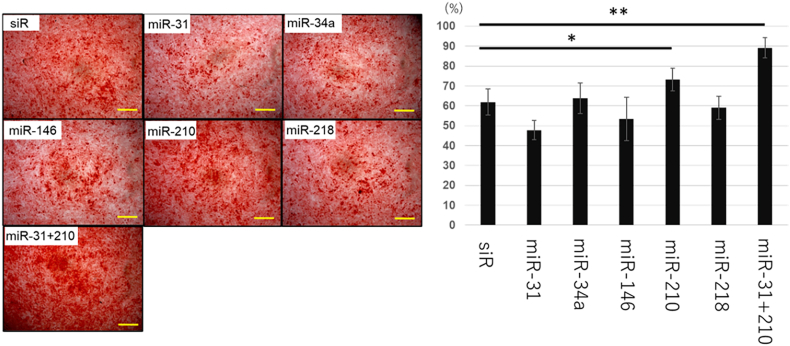
Alizarin red staining ∗: p < 0.05, ∗∗: p < 0.01.

The groups transfected with miR-210 and miR-31 + 210 exhibited significantly increased calcified areas compared to that in the siRNA group (Fig. 4).

#### 3.1.2 Tube formation of HUVECs

**Fig. 5:**
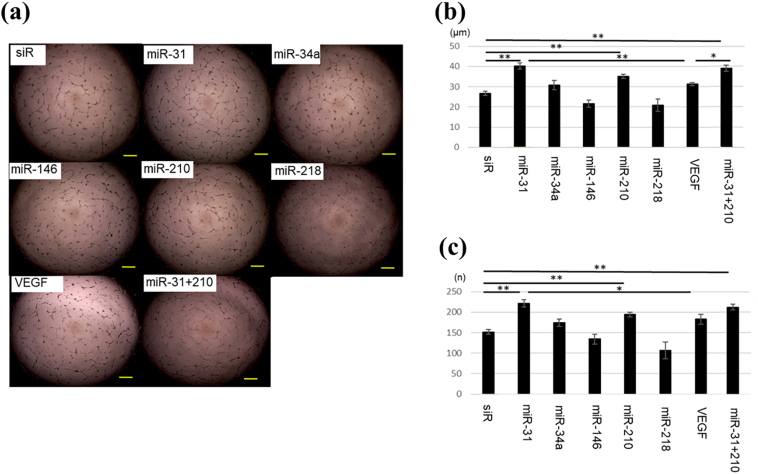
Tube formation of HUVECs ∗: p < 0.05, ∗∗: p < 0.01.

The branch points in the groups treated with miR-31, miR-210, and miR-31 + 210 were significantly higher than those in the siRNA-treated group. Furthermore, the groups treated with miR-31 and miR-31 + 210 exhibited significantly more branch points than those in the VEGF group. The total tube length in the groups treated with miR-31, miR-210, and miR-31 + 210 was significantly longer than that in the siRNA-treated group. Notably, only the group treated with miR-31 demonstrated a significantly longer total tube length than that of the VEGF group (Fig. 5).

#### 3.1.3 Quantitative RT-PCR

**Fig. 6:**
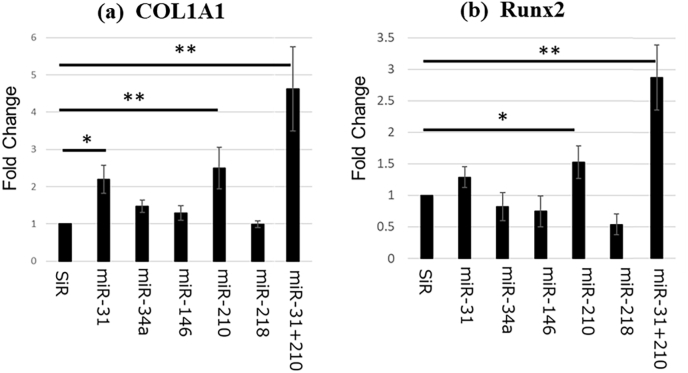
Quantitative real time polymerase chain reaction ∗: p < 0.05, ∗∗: p < 0.01.

The groups transfected with miR-31, miR-210, and miR-31 + 210 showed a marked increase in COL1A1 expression. Furthermore, these groups were associated with increased Runx2 expression compared with the siRNA group. Notably, the miR-31 + 210 transfected group showed significant osteogenic potential (Fig. 6).

#### 3.1.4 Identification of miRNAs exhibiting the greatest osteogenic and angiogenic potential

In the in vitro study, miR-210 was identified as being predominantly involved in osteogenesis, whereas miR-31 was associated with angiogenesis. Moreover, miR-31 + 210 demonstrated the most pronounced effect on bone formation, thereby warranting its selection for local administration in subsequent in vivo experiments.

### 3.2 In vivo study

#### 3.2.1 Histological analysis

**Fig. 7:**
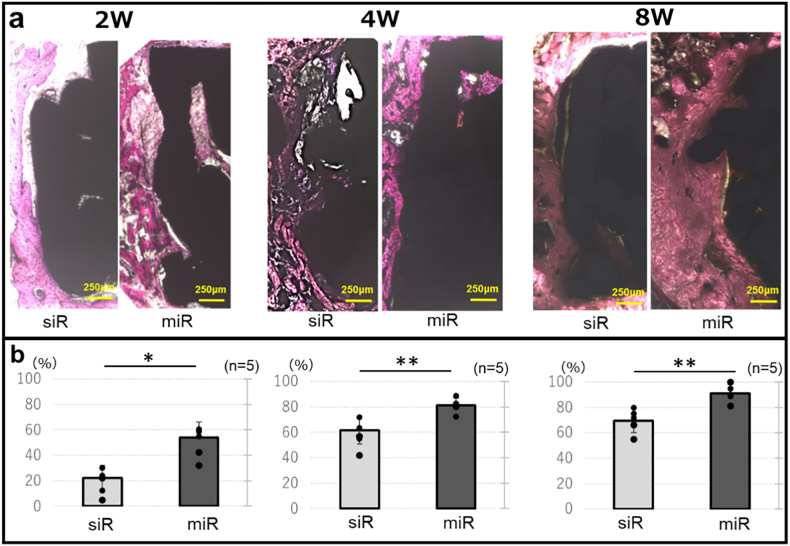
Histological analysis ∗: p < 0.05, ∗∗: p < 0.01.

In the BIC analysis, the miR group exhibited significantly higher values than those in the siR group at all evaluated time points: 2 weeks (miR group: 54.3 ± 12.2, siR group: 22.1 ± 9.9, p = 0.04), 4 weeks (miR group: 81.1 ± 5.9, siR group: 61.7 ± 11.0, p < 0.01), and 8 weeks (miR group: 91.0 ± 7.1, siR group: 69.5 ± 9.6, p < 0.01) (Fig. 7).

#### 3.2.2 Quantitative RT-PCR

**Fig. 8:**
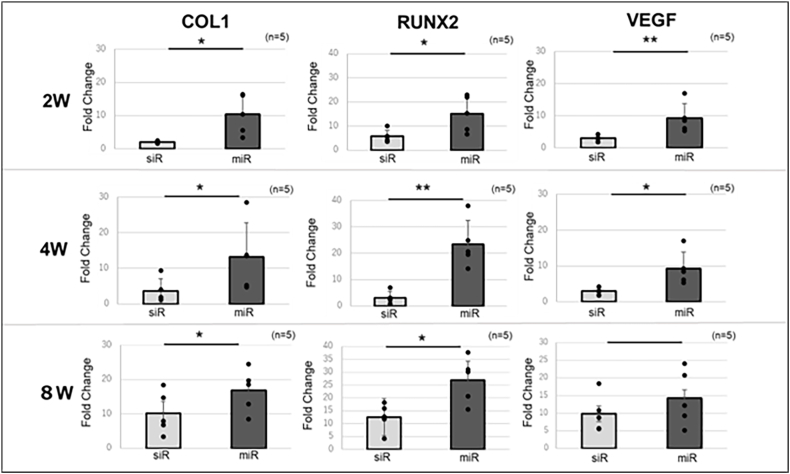
Expression levels of COL1A1, Runx2, and VEGF at each time point were determined using quantitative real-time polymerase chain reaction. ∗: p < 0.05, ∗∗: p < 0.01.

The expression levels of COL1A1 and Runx2 were significantly elevated in the miR group compared to those in the siR group at all time points. In addition, a significant increase in VEGF levels was observed in the miR group at 2 and 4 weeks; however, this significant difference was not sustained at 8 weeks (Fig. 8).

#### 3.2.3 Radiological analysis

Four weeks post-OVX, micro-CT analysis of the distal femur revealed a significant decrease in BV/TV in the OVX group compared to the sham group (OVX group: 29.7 ± 2.2 %, sham group: 46.7 ± 7.3 %, p = 0.03), confirming the successful induction of osteoporosis following ovariectomy.

**Fig. 9:**
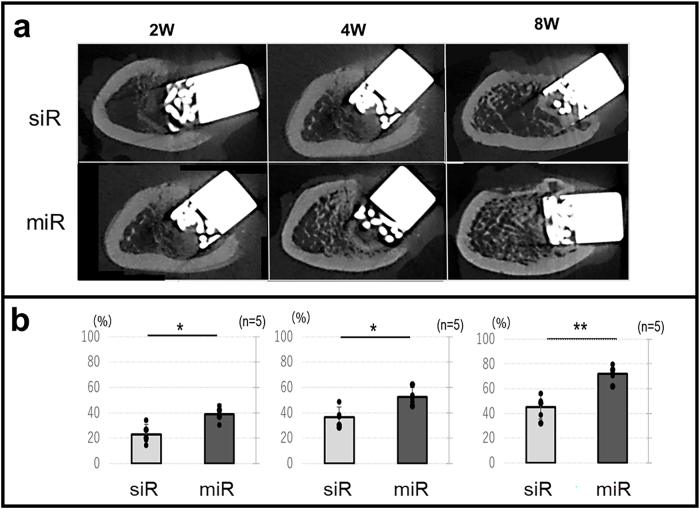
Radiological analysis ∗: p < 0.05, ∗∗: p < 0.01.

After implantation, the miR group exhibited significantly higher BV/TV than that of the siR group at all assessed time points: 2 weeks (miR group: 38.7 ± 5.8, siR group: 22.8 ± 7.7, p = 0.02), 4 weeks (miR group: 52.3 ± 6.3, siR group: 36.6 ± 7.9, p = 0.0), and 8 weeks (miR group: 72.1 ± 6.5, siR group: 45.2 ± 9.5, p = 0.02) (Fig. 9).

#### 3.2.4 Biomechanical analysis

**Fig. 10:**
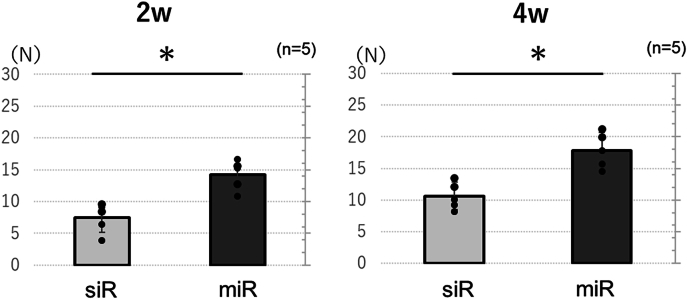
Biomechanical analysis ∗: p < 0.05.

In the biomechanical analysis, the miR group exhibited significantly greater maximum pull-out force compared to that of the siR group at all assessed time points: 2 weeks (miR group: 14.2 ± 2.3, siR group: 7.5 ± 2.4, p = 0.04), 4 weeks (miR group: 17.8 ± 2.7, siR group: 10.6 ± 2.1, p = 0.03) (Fig. 10).

## 4 Discussion

In this in vitro study demonstrated that among the miRNAs examined (miR-31, miR-34a, miR-146, miR-210, and miR-218), only miR-31 and miR-210 showed consistent enhancement of osteogenic differentiation, mineralization, and angiogenic signaling. Furthermore, the simultaneous administration of miR-31 and miR-210 resulted in the highest osteogenic potential among all tested miRNAs in the in vitro study. Other miRNAs did not produce comparable osteogenic or angiogenic effects under the same experimental conditions, leading us to select the miR-31/210 complex for in vivo study. In a subsequent in vivo study, the localized administration of miR-31 + 210 at the matrix bone of hydroxyapatite HA/TCP-coated titanium implants significantly enhanced osseointegration and improved early implant fixation. Histological analysis demonstrated increased bone formation at the implant site, with a higher BIC ratio than that in the control group at all time points. Genetic analysis revealed the upregulation of key osteogenic and angiogenic markers, including RUNX2, COL1A1, and VEGF, indicating enhanced osteoblast activity and vascularization. Radiological analysis confirmed an increase in BV/TV around the implant, suggesting improved bone matrix retention. Biomechanical testing further demonstrated that the miR-31 + 210 combination significantly improved the pull-out strength of the implant, confirming the enhanced mechanical stability. These findings indicate that the localized administration of miR-31 + 210 to the bone matrix may enhance osteogenesis and angiogenesis in HA/TCP -coated titanium implants. This approach can potentially improve the initial fixation strength of the implant and mitigate the reduction in bone mass associated with osteoporosis.

HA-coated implants demonstrated excellent osseointegration and initial fixation. Several clinical studies have reported high long-term survival rates and significant improvements in clinical scores [[33], [34], [35]]. However, HA-coated implants can inhibit osseointegration in the presence of osteoporosis, suggesting that these advantages could be restricted [36]. Furthermore, previous studies have demonstrated that the combined application of angiogenic and osteogenic proteins is more effective in promoting bone formation around implants than the use of osteogenic proteins alone, which is consistent with our in vitro study [[37], [38], [39]]. Nevertheless, while these combined proteins successfully enhanced bone mineral density (BMD), they did not facilitate osseointegration of the implant. Although implant surface processing has been considered, the specific details remain unclear [38]. However, in the present study, the combined effects of osteogenic and angiogenic miRNAs were found to enhance the osseointegration of implants. This enhancement was partly attributed to the high affinity between HA and miRNAs. Notably, the presence of HA has been reported to significantly improve the delivery of miRNAs to osteoblasts [40]. This enhancement is attributed to the high surface charge and ion-exchange capacity of HA, which facilitates electrostatic adsorption and gradual release of miRNA complexes at the implant–bone interface. In addition, the physiological dissolution of HA releases calcium and phosphate ions, inducing the formation of a carbonate apatite layer analogous to natural bone minerals, which further promotes osteoblast adhesion and differentiation on the implant surface [41]. Moreover, because the implant surface used in this study consisted of an HA/TCP composite coating, the contribution of TCP should also be considered. TCP has a substantially higher solubility than HA, leading to more rapid calcium and phosphate ion release under physiological conditions. This accelerated ion release promotes early apatite nucleation and osteoblast activation, which may support the early phase of osteogenesis. Additionally, the bioactive surface generated by TCP dissolution may enhance the adsorption and cellular uptake of miRNA complexes [42]. Therefore, the combined properties of both HA and TCP may have synergistically contributed to the improved both the early onset and sustained progression of osseointegration observed in this study.

Our findings are consistent with previous reports, indicating that miR-31 and miR-210 promote osteogenesis and angiogenesis. MiR-31 has been shown to contribute to microvesicle-triggered angiogenesis by downregulating the factor-inhibiting HIF-1 (FIH-1) in vascular endothelial cells [18]. Meanwhile, MiR-210 has been reported to promote BMP4-induced osteoblastic differentiation by inhibiting the transforming growth factor beta (TGF-β)/activin signaling pathway [21]. Furthermore, miR-210 has been recognized as a crucial regulator of postmenopausal osteoporosis. It plays a role in facilitating osteoblast differentiation, improving the microstructure of bone tissue in OVX rats, regulating bone formation and resorption, and promoting osteoblast differentiation [43,44]. Furthermore, an interactive regulation exists between the Bone Morphogenetic Protein (BMP) and VEGF signaling pathways during osteoclast formation, indicating their close relationship [45]. Although other candidate miRNAs investigated in this study (miR-34a, miR-146a, and miR-218) have been documented to exert osteogenic effects [19,20,22], their influence on angiogenesis is recognized as inhibitory. For instance, miR-34a serves as a positive regulator of osteoblast differentiation while acting as a negative regulator of HUVEC proliferation and angiogenesis [19]. Similarly, both miR-146a and miR-218 have been demonstrated to directly modulate endothelial cell function and suppress angiogenic activity [46,47]. These inhibitory effects on angiogenesis may have contributed to their limited impact in our in vitro experiments. Therefore, it is suggested that the HA/TCP-mediated delivery of miR-31 and miR-210 may have created a synergistic osteogenic–angiogenic microenvironment, in which enhanced miRNA uptake by HA/TCP to osteoblast potentially activated the BMP4 and VEGF signaling cascades, thereby contributing to accelerated osteoblast differentiation, neovascularization, and bone–implant integration.

The findings of this study highlight the potential of miRNA-based therapies with osteogenic and angiogenic effects to enhance early implant fixation and improve bone loss surrounding implants, particularly in patients with compromised bone quantity due to osteoporosis. Conventional osteoporosis medications, such as bisphosphonates and teriparatide require time to exert localized effects on bone mass and quality. In contrast, local delivery of miR-31 and miR-210 at the implant-bone interface offers a targeted and immediate approach to improve osseointegration and mechanical fixation in the early postoperative period. In the context of an aging population, where the incidence of osteoporosis among patients undergoing TJA is considerable [9,10], this suggests the necessity for novel treatment strategies beyond the HA/TCP coating of implants. Recently, advancements in technologies for coating HA or TCP with miRNAs and their applications have been reported [23,[48], [49], [50]]. These advancements are expected to facilitate the development of bioactive biomaterials for clinical use. However, research on the application of miRNAs to enhance implant fixation strength remains limited, and further studies are warranted to clarify their long-term effects.

This study has some limitations. First, the sample size was relatively small, and the observation period was limited to eight weeks, potentially reducing the statistical power to detect subtle differences and adequately assess the long-term effects of miRNA-based therapies on implant survival. Second, the precise molecular mechanisms underlying the synergistic effects of miR-31 and miR-210 remain to be fully elucidated. Third, the study lacks comparisons with other miRNAs reported to promote osteogenesis (e.g., miR-26a and miR-21), which were not used in the current experiments [51,52]. Future comparative studies are necessary to determine the optimal miRNA combinations for enhancing implant osseointegration. Fourth, our in vivo study did not include separate groups administered with miR-31 or miR-210 alone. Although our in vitro studies demonstrated that the combination of miR-31 and miR-210 exhibited the highest osteogenic potential, future studies are warranted to directly compare individual miRNA group in vivo and confirm their specific role and true synergistic effects. Last, this study was conducted using an ovariectomized rat model, which may not fully replicate the clinical conditions associated with human osteoporosis. Further research is required to investigate the long-term effects, optimize the delivery method, and assess the clinical applicability of miRNA-based regeneration therapies to enhance implant fixation in patients with compromised bone quantity.

## 5 Conclusion

Concurrent administration of miR-31 and miR-210 at the implant-bone interface markedly improved osseointegration and initial implant fixation in an osteoporotic rat model. These findings indicate that miRNA-based regeneration therapies may constitute a novel and effective approach for enhancing early implant stability, thereby mitigating the risk of aseptic loosening and improving long-term clinical outcomes following TJA.

## Ethics approval and consent to participate

This study was approved by the Ethics Committee for Experimental Animals of Hiroshima University.

## Consent for publication

Not applicable.

## Author contribution

All authors contributed to the conception and design of this study. Material preparation, data collection, and analyses were performed by SU, TS, and HS. The first draft of the manuscript was written by SU, and HK., HM., and YK contributed to the data interpretation, while NA supervised the research. All the authors have read and approved the final version of the manuscript.

## Funding

This work was supported by a grant-in-aid for Science Research from the Japanese Society for Replacement Arthroplasty Foundation.

## Declaration of competing interest

The authors declare that there are no known competing financial interests or personal relationships that could have appeared to influence the work reported in this paper.

## Data Availability

Data supporting the findings of this study are available upon reasonable request from the corresponding author.
